# Vocal Identity Recognition in Autism Spectrum Disorder

**DOI:** 10.1371/journal.pone.0129451

**Published:** 2015-06-12

**Authors:** I-Fan Lin, Takashi Yamada, Yoko Komine, Nobumasa Kato, Masaharu Kato, Makio Kashino

**Affiliations:** 1 NTT Communication Science Laboratories, NTT Corporation, Atsugi, Kanagawa, Japan; 2 Medical Institute of Developmental Disabilities Research, Showa University, Tokyo, Japan; 3 ATR Brain Information Communication Research Laboratory Group, Kyoto, Japan; 4 Center for Baby Science, Doshisha University, Kyoto, Japan; 5 Department of Information Processing, Interdisciplinary Graduate School of Science and Engineering, Tokyo Institute of Technology, Yokohama, Kanagawa, Japan; 6 CREST, JST, Atsugi, Kanagawa, Japan; Hamamatsu University School of Medicine, JAPAN

## Abstract

Voices can convey information about a speaker. When forming an abstract representation of a speaker, it is important to extract relevant features from acoustic signals that are invariant to the modulation of these signals. This study investigated the way in which individuals with autism spectrum disorder (ASD) recognize and memorize vocal identity. The ASD group and control group performed similarly in a task when asked to choose the name of the newly-learned speaker based on his or her voice, and the ASD group outperformed the control group in a subsequent familiarity test when asked to discriminate the previously trained voices and untrained voices. These findings suggest that individuals with ASD recognized and memorized voices as well as the neurotypical individuals did, but they categorized voices in a different way: individuals with ASD categorized voices quantitatively based on the exact acoustic features, while neurotypical individuals categorized voices qualitatively based on the acoustic patterns correlated to the speakers' physical and mental properties.

## Introduction

Faces and voices are the most important social stimuli in the visual and auditory domains. Faces and voices can convey information about the speakers, including identity, gender, emotional state, and age. It has been proposed that individuals with autism spectrum disorder (ASD) might process faces in a different way, and a deficit in face recognition might lead to a deficit in social interaction and communication. For example, individuals with ASD tend not to look at the eye region of faces, therefore their processing of the eye region is less effective than that of neurotypical (NT) individuals [1]. In addition, individuals with ASD are found to perform worse than NT individuals in face memory tasks [2]. Because both faces and voices are processed in partially overlapping brain areas (in the superior temporal sulcus) and have similar developmental tracks, it has been suggested that facial and vocal information might be processed in similar functional architectures so that the multi-modal information can be integrated to extract information related to personality and emotion [3].

The aim of this study was to verify how individuals with ASD recognize and memorize vocal identity. Vocal identity recognition is not trivial. Voices carry information about the vocal tract and vocal folds, which reveals the gender and age of the speaker [4]. In addition to these anatomically defined factors, voices are modulated by less permanent factors such as emotion and health [5, 6]. In everyday life, we recognize people's voices independently of the content of their speech or their emotion. It is interesting to consider how our vocal identity recognition system can extract relevant features from acoustic signals that are invariant to the modulation of those acoustic signals and form the acoustic patterns correlated to the speakers' physical and mental properties [7, 8].

Research into voice perception in ASD has shown that infants with ASD have different listening preferences as regards their mother's speech [9], and children with ASD take longer time to orient to social stimuli than NT children [10]. In addition, the cortical activation patterns evoked by vocal sounds (including speech and non-speech sounds) and complex voice-like sounds in individuals with ASD are found to be less significant than in NT individuals in the voice-selective superior temporal sulcus area [11–13]. Children with ASD also exhibit underconnectivity between the voice-selective cortex and reward pathways such as the dopaminergic reward pathway and the amygdala-related association learning system [14]. Nevertheless, individuals with ASD are found to perform similarly to NT individuals in discriminating gender in voice [15]. Research into vocal identity recognition has brought mixed results [16, 17], and research investigating emotion recognition in voices has also produced mixed findings [17–19].

In this study, we adapted an experimental procedure that was used to evaluate phonagnosia quantitatively [20]. This study first evaluated gender discrimination, and then it evaluated how listeners memorized the connection between names and their corresponding voices. Finally, this study evaluated the way in which listeners differentiated voices learned in the previous experiments and brand new voices. The speech corpus we used provides single words spoken with neutral emotion, and the participants did not hear these voices before this study. In the experiments, the speakers always uttered different words, so the listeners needed to extract the vocal identity information while the speech content varied. Because single words were used instead of full sentences, there was less temporal information such as prosody.

## Methods

### Participants

Fourteen NT individuals (ranging in age from 20 to 43, 3 females) and 14 individuals with ASD (ranging in age from 20–47, 3 females) participated in this study. Written consent from the patients and NT individuals was gathered before we conducted the experiments. All procedures were conducted in accordance with the Declaration of Helsinki and approved by the Ethics Committee of the NTT Communication Science Laboratories. All of the participants had normal hearing and were naive to the purposes of the study. The participants were paid for their time.

The diagnosis of ASD was based on a consensus reached by three experienced psychiatrists and one psychologist according to the criteria of Diagnostic and Statistical Manual of Mental Disorders (DSM-IV-TR) [21], after two detailed interviews conducted independently by a psychiatrist and a clinical psychologist belonging to the team at Karasuyama Hospital that included the participant's developmental history, present illness, past history, and family history. Of the 14 ASD participants, 6 were diagnosed with Asperger's syndrome (AS), 5 were diagnosed as having high-functioning autism (HFA), and 3 were diagnosed as exhibiting Pervasive Developmental Disorder-Not Otherwise Specified (PDD-NOS). None of the participants met the DSM-IV-TR criteria for any other psychiatric disorders.

All the participants took a WAIS-R or WAIS-III IQ test (FIQ mean±SD NT group: 113.14±9.18, ASD group: 108.21±11.21; VIQ mean±SD NT group: 119±9.74, ASD group: 112.93±11.01; PIQ mean±SD NT group: 102.64±12.32, ASD group: 100.57±12) and were evaluated regarding their autistic traits by autism spectrum quotient except one NT participant (mean±SD NT group: 17.85±5.65, ASD group: 36.36±5.11). There was no significant difference between the ages and IQ scores of these two groups, but these two groups had significantly different autistic traits (t(25) = 8.93, p<0.001).

### Auditory stimuli

The auditory stimuli used in the experiment were selected from the speech corpus 'Japanese phonetically-balanced word speech database' (ETL-WD) produced by the National Institute of Advanced Industrial Science and Technology and published by the National Institute of Informatics Speech Resources Consortium in Japan. The auditory stimuli were originally recorded (and played back) with a 16000-Hz sampling rate. Their spoken words were meaningful and consisted of two or three syllables with neutral emotion. We selected speech materials from 10 female and 10 male speakers. All the presented spoken words were shorter than 1.125 s, and speech signals longer than 1.125 s were not selected. The averaged duration of the voices in the training dataset was 0.65 s, and the standard deviation was 0.15 s.

The experiment was conducted in a sound-insulated booth. Auditory stimuli were processed by an audio interface (M-AUDIO FAST-TRACK PRO) and then sent to headphones (Senheiser HDA200). The auditory stimuli were presented at a comfortable sound level for individual participants.

### Procedures

This study comprised three experiments: (1) gender discrimination, (2) vocal identity recognition via naming, and (3) familiarity test. Among the 10 female and 10 male speakers, speech produced by 5 female and 5 male speakers was selected to be presented in the first and second experiments. These 10 training speakers were selected to provide sufficient discriminability based on a pilot experiment. Speech produced by all 20 speakers was presented in the third experiment. The spoken words were randomly selected for each participant. Participants were instructed to respond as accurately as they could.

(1) Gender discrimination: Participants listened to speech produced by 10 selected speakers (10 spoken words from each speaker) and gave their answers regarding the gender of the speakers by clicking the male/female figures presented on the computer screen. In total, listeners heard 100 spoken words that were presented in a random order. No feedback was provided.

(2) Vocal identity recognition via naming: In this part of the experiment, the participants were trained and tested separately for vocal identity recognition of female and male speakers. This experiment consisted of 6 training and 6 test sessions for speakers of each gender. In each session, 10 spoken words were presented from each speaker. A training session was always followed by a test session with the same speakers. The session order with female or male speakers was selected randomly for individual participants.

In the training sessions, five names corresponding to individual speakers were presented on the computer screen. In each trial, a name was underlined in red to indicate the identity of the speaker who had produced the spoken word. Participants were instructed to remember the correspondence between the speakers’ names and their voices. In the test sessions, the same five names were presented on the computer screen. In each trial, after the participants heard a spoken word, they used the mouse to click the name of the speaker presented on the screen who had produced that word. The presentation order of the spoken words in each session was randomly arranged. The correct answer was underlined in red once the participants gave their answer.

(3) Familiarity test: 200 spoken words produced by 20 speakers (10 spoken words from each speaker) were presented in a random order. In each trial, the participants used the mouse to click 'familiar' presented on the screen if they had heard that speaker during the first two experiments and click 'unfamiliar' presented on the screen if they had not heard that speaker before. No feedback was provided.

## Results

The following statistic analyses were conducted in two ways: In one way, the results for the ASD group were pooled together, and in another way, the results for the participants diagnosed as AS and the results for the participants diagnosed as HFA were processed separately while the results for the participants diagnosed as PDD-NOS were not included due to the small sample size (N = 2). The ANOVA analysis was conducted with SPSS v.19 (IBM, USA).

### Gender discrimination experiment

The performance of both the ASD and NT groups reached the ceiling level (the average percent correct was 96.5% and 98.9% for the ASD and NT groups, respectively). A t-test comparing the d-prime of gender discrimination (calculated from the percent correct and false positive rate for unbiased measurement of the performance) for the participants in these two groups confirmed that their performance did not differ significantly (t(26) = 1.53, p = 0.138).

A one-way ANOVA was conducted on their d-prime with Group (with three levels: AS, HFA, and NT) as the between-subject factor. Results showed that Group was a significant factor (F(2,22) = 5.88, p = 0.009). However, the post hoc analysis (Games-Howell test) showed no significant difference between groups. (Games-Howell test was used because Levene test showed significant difference in the variance of d-prime in the three groups (p<0.001).)

### Vocal identity recognition via naming

**Fig 1:**
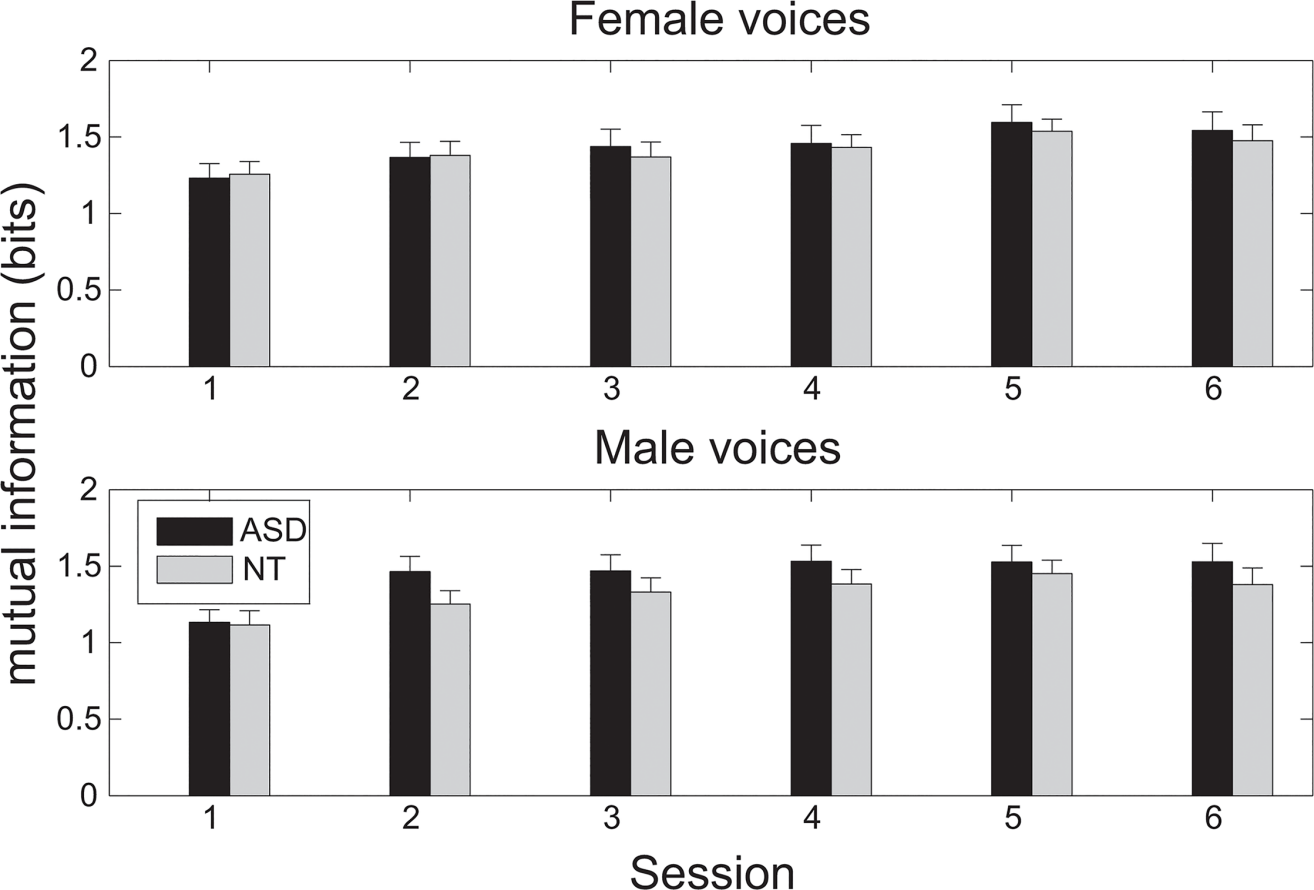
Listeners' performance in the experiment of vocal identity recognition via naming. Their performance was evaluated for female voices (above) and for male voices (below) across 6 sessions, indicated as mean ± standard error. There was significant improvement across sessions, but there was no significant difference between the ASD group and the control group. Performance was evaluated in terms of mutual information, which was calculated based on five test speakers.

Information theory was applied to provide an unbiased measurement of vocal identity discrimination. Mutual information was used to estimate how certain the listeners gave their answer based on what they heard: High mutual information indicates low uncertainty, and vice versa. Mutual information was calculated in each test session based on a 5-by-5 confusion matrix (for five speakers of each gender): The stimuli entropy was added to the responses entropy, and the joint entropy of the stimuli and responses was subtracted from the previous summation of entropies. To investigate whether the learning curve differed in different groups for speakers of different genders, the mutual information in 6 sessions for speakers of each gender (Fig 1) was analyzed by using a three-way mixed design ANOVA, with Group (with two levels: ASD and NT) as the between-subject factor and Gender and Session as the within-subject factors. The results of the ANOVA analysis revealed the significant effect of Session (F(3.382, 87.931) = 18.05, p<0.001, with Greenhouse-Geisser correction) but not Group (F(1,26) = 0.408, p = 0.529) or Gender (F(1,26) = 1.24, p = 0.276) or interactions between these factors.

Another two-way ANOVA was conducted for their mutual information with Group (with three levels: AS, HFA, and NT) as the between-subject factor and Gender and Session as the within-subject factor. Similar to the previous ANOVA analysis with two levels in the factor as Group, the results revealed the significant effect of Session (F(5,110) = 11.418, p<0.001) but not Group (F(2,22) = 2.243, p = 0.13) or Gender (F(1,22) = 1.035, p = 0.32) or interactions between these factors.

### Familiarity test

**Fig 2:**
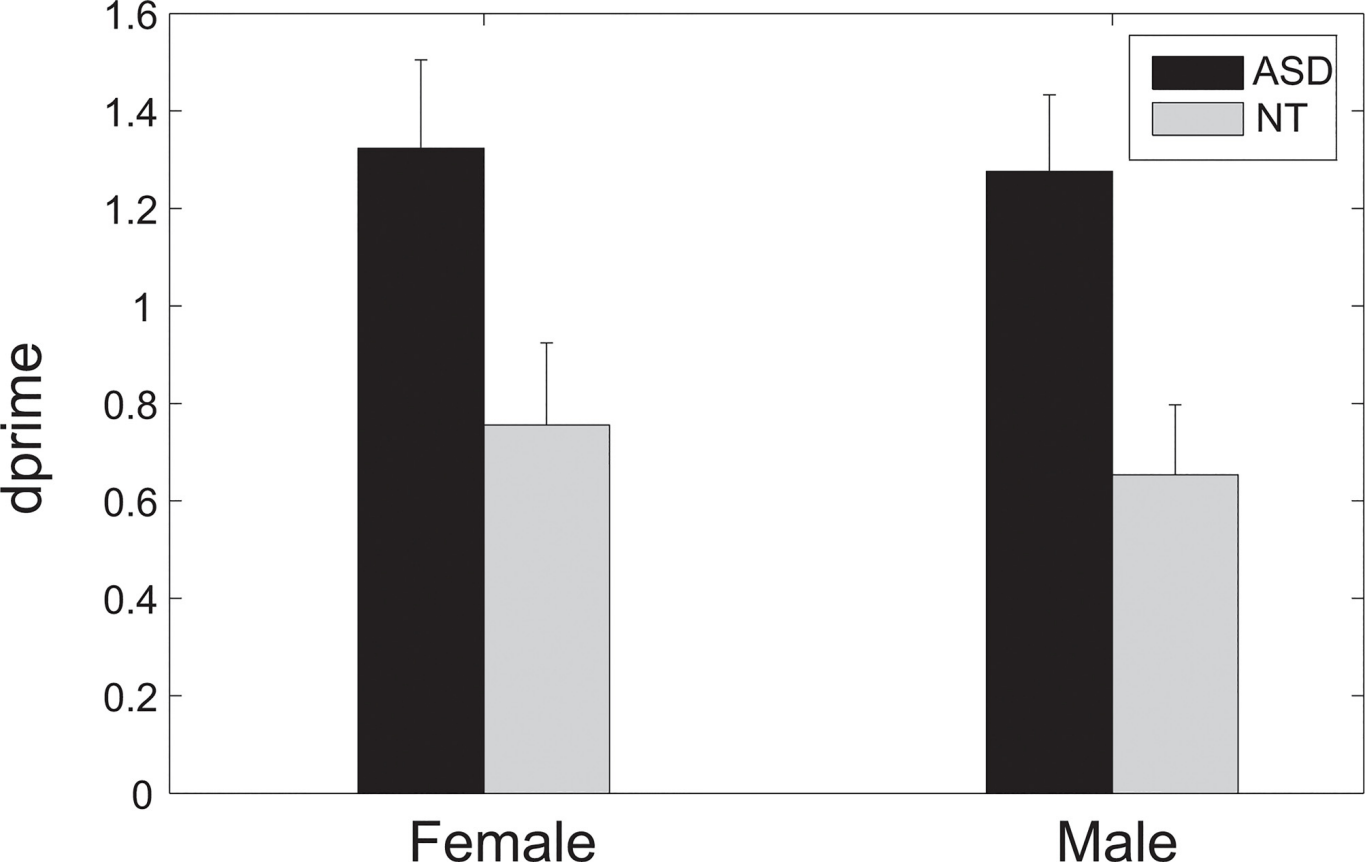
Listeners' performance in the familiarity test. Their performance was evaluated for female voices and male voices, indicated as mean ± standard error. The performance, evaluated in terms of d-prime, was significantly better in the ASD group than in the control group.

The average d-prime (calculated from the percent correct and false positive rate for the unbiased measurement of the performance) across subjects was 1.32 for the female speakers and 1.28 for the male speakers in the ASD group and 0.76 for the female speakers and 0.65 for the male speakers in the NT group (Fig 2). To investigate whether the familiarity discrimination differed for speakers of different genders in different groups, a two-way mixed design ANOVA, with Group (with two levels: ASD and NT) as the between-subject factor and Gender as the within-subject factor, was performed on their d-prime. The results of the ANOVA revealed a significant effect of Group (F(1,26) = 7.421, p = 0.011) but not Gender (F(1,26) = 0.983, p = 0.33) or the interaction between Group and Gender (F(1,26) = 0.131, p = 0.72). To investigate whether this between-group difference in d-prime came from the percent correct or false positive rate, another two two-way mixed design ANOVAs, with the between-subject factor as Group and the within-subject factor as Gender, were performed for their percent correct and false positive rate. The ANOVA results for percent correct revealed a significant effect of Group (F(1,26) = 12.509, p = 0.002) but not Gender (F(1,26) = 1.958, p = 0.174) or the interaction between Group and Gender (F(1,26) = 0.976, p = 0.332). On the other hand, the results of the ANOVA for the false positive rate revealed no significant effect of Group (F(1,26) = 0.507, p = 0.483) or Gender (F(1,26) = 2.243, p = 0.146) or the interaction between Group and Gender (F(1,26) = 0.043, p = 0.837).

Another two-way ANOVA was conducted on their d-prime with Group (with three levels: AS, HFA, and NT) as the between-subject factor and Gender as the within-subject factor. Similar to the previous ANOVA analysis with two levels in the factor as Group, the results revealed a significant effect Group (F(2,22) = 4.398, p = 0.025) but not Gender (F(1,22) = 0.267, p = 0.61) or the interaction between Group and Gender (F(2,22) = 0.291, p = 0.75). Post hoc test (with Bonferroni correction) shows a significant difference between the d-prime in the AS group and in the control group (p = 0.023), but there was no significant difference between the AS group and the HFA group or the HFA group and the control group (p = 0.185 and 1, respectively).

## Discussion

This study investigated how individuals with ASD recognized and memorized vocal identity. The results show that adults with ASD did not exhibit worse performance than NT adults in vocal identity recognition in a naming or familiarity test as in the case report of developmental phonagnosia [20]. Compared with the control group, the ASD group performed similarly in gender discrimination and vocal identity recognition via naming, and they performed better in the familiarity test. Although the statistic analysis showed that the participants with Asperger's syndrome but not the participants with high-functioning autism performed significantly better than the control group in the familiarity test, it is not conclusive due to the small sample size in each patient group.

The discrimination of vocal gender is mainly based on the fundamental frequency and resonant frequencies (i.e., formants) and spectral envelope [22]. In our gender discrimination experiment, none of these acoustic cues were modified. Therefore, the participants in this experiment could easily reach the ceiling level if they were able to use these cues. Although our comparison of the performance of the ASD group and the performance of the control group in this experiment suffered from the ceiling effect, our finding that there was no significant difference between the two groups is consistent with a previous study, in which the gender of the voice fragments was parametrically morphed by shifting the formant ratio and median pitch of the male and female voices [15].

Although we are not aware of any clinical report about phonagnosia in ASD, we cannot rule out the possibility that developmental phonagnosia is difficult to notice [20]. Based on the imaging studies [13, 14] that reveal different brain activation patterns when individuals with ASD and NT individuals hear human vocal sounds, it is supposed that some aspects of voice perception are affected in individuals with ASD. Nevertheless, previous studies investigating emotion recognition in the voice had mixed findings [17–19], as did previous studies investigating vocal identity recognition [16, 17]. The results of our second experiment show that individuals with ASD were able to extract relevant features from acoustic signals to form categories that are invariant to the modulation of acoustic signals as well as NT individuals. Moreover, the individuals with ASD outperformed NT individuals in our third experiment, the familiarity test.

At first glance, the superior performance observed in the familiarity test in the ASD group is surprising because it contrasts with the findings of previous studies with similar design [16, 17]. However, it is not so surprising if we consider the difference in the speech materials used in these studies is considered. To be specific, in previous studies, each speaker uttered a much longer sentence, which contains rich temporal information such as prosody, speech rhythm, and accent to distinguish vocal identity. On the other hand, in this study, participants only heard speech that contained one word with only two or three syllables with neutral emotion, so listeners in this study might not be able to use the rich temporal cues in voices to recognize the identity of each voice. Instead, they might need to utilize the spectral information in the voices such as the fundamental/formant frequencies, pitch contours, and the shape of spectral envelope [23, 24]. It has been found that compared to NT individuals, individuals with ASD have worse receptive prosodic skills [25] but increased sensitivity to musical timbre [26]. Therefore, compared to the control group, the ASD group might perform worse in vocal identity recognition if the prosodic information can be extracted from spoken sentences, and the ASD group might perform better if the timbre cues (e.g., formant frequencies and the spectral envelope) in the voices are the major source for vocal identity recognition.

Although the second and the third experiments both investigated voice memory, they probed different aspects in voice that are stored in memory. To be specific, the second experiment examined how participants formed and memorized the category of voices, and the third experiment examined how they re-categorized voices when exposed to new voices. If the ASD group and the control group adopted the same ways to memorize the voices but the ASD group had better memory than the control group had, they should have superior performance in both the second and third experiments. On the other hand, if the ASD group and the control group adopted different ways to memorize the voices, the ASD group might have normal performance in the second experiment but superior performance in the third experiment.

In the familiarity test, the performance with NT individuals was comparable to that reported in previous studies [20, 27], but individuals with ASD exhibited superior performance. If voices are categorized qualitatively based on their acoustic patterns related to the speakers' physical and mental properties (e.g., in terms of hoarseness, harshness, and hypernasality, or even dark, bluesy, and thick), the category of voices is formed through comparison and the boundaries are adaptable. On the other hand, if voices are categorized in a quantitative way based on their exact acoustic features (e.g., formant frequencies and spectral envelope), the category of voices is formed before the exposure to any stimulus and the boundaries are rigid. For example, in the third experiment in which new voices were mixed with old voices, if participants categorized voices qualitatively, they would have difficulty in differentiating new voices from old voices because they did not have the chance to evaluate the difference between the new and old voices qualitatively to form new category. On the other hand, if participants memorized the exact acoustic features in the old voices, their performance should be superior because they could tell the difference between the new voices and old voices by putting the new voices and old voices in the strictly defined category. In a previous study, individuals with ASD did not exhibit any facilitation of discrimination near the category boundary compared with NT individuals [28]. These researchers argue that the absence of the influence of categorical knowledge reflects an increased low-level process and reduced top-down influence. If their argument is true, the superior performance in the familiarity test found in the ASD group should not be simply attributed to their superior performance in discriminating auditory stimuli based on different acoustic features. Instead, it is possible that individuals with ASD used a different way to form their categorical memory for voices based on the exact acoustic features in the voices, and this strategy was responsible for the superior performance in the familiarity test found in the ASD group.In summary, individuals with ASD were able to use acoustic features to differentiate gender and identity information from voices that varied in terms of speech content. Their performance was comparable to that of NT individuals as regards gender discrimination and identity recognition via naming, and their performance was better than NT individuals’ performance with respect to the familiarity test. Considering the speech corpus used in this study, their superior performance might be attributed to their special way to form categorical memory based on the exact acoustic features in voices. Our findings show the potential to facilitate voice perception in individuals with ASD via acoustic cues.
